# Emotional Labor in Teaching Chinese as an Additional Language in a Family-Based Context in New Zealand: A Chinese Teacher’s Case

**DOI:** 10.3389/fpsyg.2022.902700

**Published:** 2022-06-15

**Authors:** Chunrong Bao, Lawrence Jun Zhang, Helen R. Dixon

**Affiliations:** ^1^School of Foreign Language Education, Jilin University, Changchun, China; ^2^Graduate School of Education, Peking University, Beijing, China; ^3^Faculty of Education and Social Work, The University of Auckland, Auckland, New Zealand

**Keywords:** emotional labor, Chinese as an additional language, family-based context, narrative inquiry, New Zealand

## Abstract

New Zealand is a multilingual and multicultural society, where English, Maori, and the New Zealand sign language are designated as its official languages. However, some heritage languages (e.g., Chinese/Mandarin Chinese, French, German, Japanese, and Korean, among others) are also taught either within or outside the national education system. During the past decade, an increasing number of students have chosen Mandarin Chinese (hereafter “Chinese”) as an additional language (CAL) because of its fast-growing importance. To date, studies regarding CAL are mainly based on the mainstream Chinese programs (i.e., in schools or universities) or online platforms, with less attention paid to other types of teaching contexts (e.g., family-based and private tutoring contexts) where there also exist many potential challenges awaiting teachers. To fill in this gap, this study, based on a teaching program consisting of two families in New Zealand, explored the trajectories of a CAL teacher’s emotional labor for 47 weeks to understand how she managed her emotions when she taught the language as well as balanced the relationship among the three parties: the institution, the two families, and herself. Narrative inquiry was used as a methodological approach. The data involved written and spoken narratives. Using inductive and deductive thematic analysis, findings revealed her different understandings of the emotional labor in the two families, respectively, during the program. Further analysis of the data revealed some factors that impacted her emotional labor and how they impacted her teaching in a family-based context. We concluded our study with a discussion of the implications of these findings for teaching CAL in similar contexts.

## Introduction

Multilingualism has become increasingly common around the world (Cenoz and Gorter, 2015; Tai and Li, 2020), especially mastery of languages of wider communication, because the ability to use these languages not only provides better opportunities (e.g., in the job market) but is also linked to the users’ identities and memberships to the individuals’ speech communities (Cenoz and Gorter, 2015; Jiang and Zhang, 2021). New Zealand is a case in point as it is mainly a country of immigrants, predominantly English-speaking people from the United Kingdom who arrived in New Zealand in its early history.^1^ However, New Zealand is now considered a multilingual and multicultural society (Kaplan, 1994), where English, Maori, and the New Zealand sign language are designated as its official languages. Some heritage languages (e.g., Chinese/Mandarin Chinese, French, German, Japanese, and Korean) are also taught. Therefore, students have the opportunity to choose one of these heritage languages as an additional language either within or outside the national education system (Wang, 2020). During the past decade, an increasing number of students have chosen Mandarin Chinese (hereafter “Chinese”) as an additional language (CAL) because of its fast-growing importance (Eriksen, 2018; Wang, 2020).

Motivated by this CAL-learning trend, some parents not only encourage their children to learn Chinese but also learn CAL together with their children. Therefore, in some CAL classes, it is not rare to find that students are from the same family, of different ages, and with diverse ethnic backgrounds. Thus, CAL teachers must not only teach students from various cultural backgrounds but also work in more diverse contexts (e.g., mainstream programs in schools or universities, community programs, family-based contexts, and private tutoring contexts). In addition, CAL teachers have to frequently rearrange teaching contents, readjust teaching strategies, reconstruct their identities, and regulate emotions to satisfy the needs of different types of classes, as noted by other studies in relation to teaching languages other than Chinese (e.g., Benesch, 2020; Alzaanin, 2021; Sun and Zhang, 2021; Wu et al., 2021; Zhang, 2021). However, there neither exists research sharing the criteria for categorizing the learning communities within a class, such as by students’ ages, their cultural backgrounds, or their individual families nor exists research on experiences telling teachers where they should stand or what their identities should be within a class with multiple cultures. All these pose many potential challenges for teachers who teach this type of class. Facing such challenges, teachers are told that their self-efficacy beliefs would work effectively (e.g., Sela-Shayovitz and Finkelstein, 2020; Bao et al., 2021) as long as they can be supported by mastery/performance experiences (i.e., personal authentic experiences), vicarious experiences (i.e., authentic experiences of other people), social persuasion, and their physiological and emotional states (Bandura, 1995, 2006).

In addition, in the field of language teaching, studies on teacher emotions are mainly in the context of teaching English as a foreign language (EFL; e.g., Kang, 2020; Alzaanin, 2021; Dewaele and Wu, 2021; Shi, 2021) rather than CAL; those available studies are based on mainstream Chinese programs (i.e., in schools or universities) or online platforms (e.g., Gao and Zhang, 2020; Bao et al., 2021; Peng and Pyper, 2021; Wang and Zhang, 2021), with less reference to different teaching contexts. To this end, this study was conducted in a family-based context in New Zealand to explore a CAL teacher’s emotional labor from her own perspective, to get a fuller understanding of how she managed her emotions when teaching the language, and of how she balanced the relationship among the three parties: the institution, the two families, and herself. The following set of research questions (RQ) was followed:

RQ1: When a teacher teaches a family from a single cultural background, what will her emotions be like? How will she manage these emotions?

RQ2: When a teacher teaches a family with multiple cultural backgrounds, what will her emotions be like? How will she manage these emotions?

RQ3: What factors have impacted the CAL teacher’s emotions and emotional labors?

## Literature Review

### Theorizing “Emotion”

“Emotion” has led to debates among the scholars in many fields (e.g., history, geography, anthropology, and sociology), and those scholars have attempted to analyze “what on earth emotion is” using various approaches (e.g., biological approaches, cognitive approaches, and post-structural/discursive approaches; Benesch, 2017). Despite the lack of agreement, these debates still have centered on the two basic accounts: the *organism* account and the *interactive* account (Hochschild, 1979). The *organism* account views emotions as biological reflections (e.g., instinct or impulse) characterized by fixity and universality (Hochschild, 1979), regardless of social factors (Hochschild, 1979). In contrast, the *interactive* account considers emotions as a psychobiological means of adaptation (e.g., thinking, perceiving, and imaging intrinsically; Hochschild, 1979), noticing the influences of social factors (e.g., social norms and cultural differences; Turner, 2000); as such, emotions typically arise in response to, and convey the meanings about, a person’s changing relationships (Mayer et al., 1999).

In educational research, “emotion” is commonly analyzed with cognitive (Benesch, 2020) and discursive approaches (Zembylas, 2005a; Benesch, 2020; Li and Liu, 2021). Within a cognitive framework, teachers’ emotions, as privately experienced psychological phenomena (Benesch, 2020), are related to teachers’ identities (Pavlenko, 2003; Park, 2012; Zhang and Zhang, 2015; Fairley, 2020), instructional practices, teacher–student relationships (Hagenauer and Volet, 2014; Zhang and Zhang, 2020), and teachers’ professional development (Song, 2018). Whether emotions contribute to predetermined outcomes (e.g., students’ learning outcomes) is a criterion by which emotions are categorized into positive and negative ones (Benesch, 2020; Wang et al., 2021). Usually, the emotions favored in teaching contexts involve confidence, optimism, passion (Dewaele, 2018), empathy, respect, trust and responsiveness (Gkonou and Mercer, 2017; Benesch, 2020), and grit (Liu and Song, 2021); therefore, teachers are encouraged to create positive rapport with their students (Dewaele et al., 2018; Benesch, 2020). Within a discursive framework, teachers’ emotions are considered to be shaped by culture, power, and ideology (Zembylas, 2005a; Corbin and Strauss, 2008; Miller and Gkonou, 2018; Benesch, 2020). Hence, teachers’ emotional management not simply involves the promotion of so-called favored emotions but also encourages emotional resistance to unjust conditions (Song, 2018; Benesch, 2020; Sybing, 2021), thereby calling for emotional labor.

### Theorizing “Emotional Labor”

Emotional labor is the work of managing emotions, or people’s negotiation of the relationship between “how they feel” and “how they are supposed to feel” in particular situations (Benesch, 2017). While negotiating, people might fake, induce, enhance, or suppress their feelings to sustain the “proper” state of mind and behavior to follow the *feeling rules* specified by society and institutions, regardless of their actual feelings (Diefendorff et al., 2005; Zembylas, 2007; Hochschild, 2012; Benesch, 2017; Benesch, 2018). Research on emotional labor is relevant to many types of careers, including service-oriented and female-dominated professions (Acheson and Nelson, 2020) such as teaching (e.g., Truta, 2014; Humphries, 2020) and nursing (e.g., Gray, 2010; Sawbridge and Hewison, 2013).

In the educational context, there are two main camps of scholars researching emotional labor (Benesch, 2017): structural research and post-structural research (hereafter “structural camp” and “post-structural camp”). The structural camp deconstructs emotional labor into several components, such as the three dimensions or strategies: surface acting (i.e., people try to experience the desired emotions by hiding felt emotions or faking required emotions), deep acting (i.e., people display the desired emotions by modifying their felt emotions so that genuine emotions naturally follow; Grandey, 2003; Diefendorff et al., 2005; Hochschild, 2012), and the expression of naturally felt emotions (i.e., the emotions people show to match what they feel spontaneously; Diefendorff et al., 2005). This camp, utilizing statistical analysis (Benesch, 2017), also looks into and measures how these components are correlated with other features, such as emotional intelligence (e.g., Yin et al., 2013; Dewaele and Wu, 2021), self-efficacy (e.g., Yin et al., 2017; Xie et al., 2022), leadership practices (e.g., Zheng et al., 2018), motivation (e.g., Truta, 2014), Confucian familism, work–family conflict, emotional exhaustion (e.g., Zhu et al., 2021), public service motivation and job satisfaction (e.g., Li and Wang, 2016). In contrast, the post-structural camp does not divide emotional labor into components; instead, it usually employs qualitative methods (Benesch, 2017) to look at emotional labor in social contexts and advocates that emotional labor is discursively constructed and reproduced through intrapersonal/interpersonal/intergroup interactions (e.g., Zembylas, 2005b; Benesch, 2017; Costa et al., 2020; Humphries, 2020).

Despite the different perspectives held by the two camps, taking one camp does not mean denying the other one; rather, the perspectives of the two camps can coexist and work together. Consider education as an example. Since teaching is a relational activity (Miller and Gkonou, 2018), teachers’ emotional labor is in the dynamics of teaching (Benesch, 2017; Miller and Gkonou, 2018; King et al., 2020; Dewaele and Wu, 2021). In this context, teachers might exercise emotional labor negatively or positively (Miller and Gkonou, 2018). From the perspective of the structural camp, whether teachers use one emotional labor strategy at a time or adopt all the three simultaneously is determined by situational variables (e.g., positive or negative display rules) and their personal dispositional variables (e.g., extraversion, neuroticism, openness, conscientiousness, agreeableness, and self-monitoring; Diefendorff et al., 2005). From the perspective of post-structural camp, usually, teachers’ positive undertakings would not only benefit their students but also bring themselves professional well-being and emotional rewards (Benesch, 2017; Gkonou and Mercer, 2018; Miller and Gkonou, 2018; Oxford, 2020). Notably, both situational variables and personal variables hinge on social contexts, without which these variables are meaningless. In this sense, this study reconciled the perspectives of the two camps: (1) Following the structural camp, this study explored how the CAL teacher exercised her emotional labor, under the guidance of the three dimensions or strategies (i.e., surface acting, deep acting, and the expression of naturally felt emotions; Diefendorff et al., 2005). Since deep acting can be considered as a transitional zone between surface acting and the expression of naturally felt emotions, this study positioned it as a “turning point” as well as a part of the expression of naturally felt emotions. (2) Following the post-structural camp, this study employed narrative inquiry, a form of qualitative method (Barkhuizen et al., 2014), to analyze how the interpersonal/intrapersonal interactions in social contexts influenced the CAL teacher’s choices of these strategies.

## This Study

### Context

This study is based on a CAL class in a local program conducted by a group of Chinese New Zealanders, where Chinese was taught once a week. Since all of the teachers were native Chinese speakers, this program attracted a large number of local Chinese learners.

Before the CAL-learning trend, students enrolled in this program were mainly from Chinese-heritage families and were able to speak Chinese at home, so they mainly aimed to learn Chinese characters and culture linked to their identities. Therefore, the textbooks used (hereafter “Textbook A”) were designed for native Chinese speakers: texts were written in Chinese characters without English explanation, occasionally with *pinyin* (i.e., the way to mark pronunciation of Chinese characters) above some Chinese characters. For example, “Lesson 1” in the textbook, rather than from daily conversations (e.g., Greetings), started from such Chinese characters with the fewest strokes as “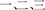 (indicating 1, 2, 3 respectively).” Therefore, it was difficult for non-native Chinese speakers to follow.

The class in this study was the first one enrolling non-Chinese-heritage students: six students from two families. For ethical reasons, all the details of their personal information were removed, with letters A and B to indicate their families (e.g., Family A and Family B). The two families also represented two typical types of New Zealand families: Family A representing a single culture, and Family B with multiple cultures (refer to Table 1). However, as the program did not prepare textbooks designed for non-native Chinese speakers, the six students had to use Textbook A during the first month.

**TABLE 1:** Information about the two families.

Family members		Age	Reasons why they came to New Zealand (hereafter “NZ”)	The countries where they came from	Mother tongue(s)	Reasons why they chose to study Chinese
Family A	Mother A	40s	She and her husband came to work in NZ	An English-speaking country	English	The children were encouraged to master more languages
	Daughter A	10	They came to NZ with their parents		English	
	Son A	7			English	
Family B	Mother B	30s	They studied in a NZ university and became NZ citizens after graduation	An Asian country	The language of her motherland	The father in this family needed to communicate with Chinese people in his business; he also hoped his wife and daughter could learn Chinese together
	Father B	30s		One of Pacific Island countries	The language of his motherland	
	Daughter B	6	She was born in NZ	NZ	English and the languages of her parents’ motherlands	

### Participant

The participant was CB (the first author of this study), the teacher who taught the class for 47 weeks. This was her first job in New Zealand. Predictably, many challenges were swamping her: as a newcomer to this country, she needed to get familiar with everything in daily life; as a Chinese citizen and new member of the program, she was an outsider to other teachers there; as a teacher of this special class, she needed to balance unpredictable relationships. Then all her previous experiences of teaching did not work effectively. However, she cherished the working opportunity very much, so she managed to survive it.

As a participant, CB reflected on her personal experiences that made her become a language teacher (De Fina, 2015; Fairley, 2020). As one of the researchers, CB analyzed her emotional states more deeply than the other two coauthors, and she interpreted her emotional labor strategies in due consideration of the reality she faced.

### Research Method

This study employed narrative inquiry (Clandinin and Connelly, 2000; Barkhuizen et al., 2014) as its methodological approach. Storytelling is regarded as one of the basic modes of thought, deeply rooted in everyday thinking (Bruner, 1986). It is also taken as a mode of understanding (Freeman, 2015), through which people construct their daily lives, daydreaming, and a sense of themselves (Freeman, 2015). Therefore, people cannot live without storytelling (Barkhuizen et al., 2014). Narrative inquiry is an approach that brings storytelling and research together through both analysis of narratives (i.e., stories used as data: moving from stories to common elements; Polkinghorne, 1995; Barkhuizen, 2015) and narrative analysis (i.e., storytelling used as a means of analyzing data and presenting findings: moving from common elements to stories; Polkinghorne, 1995; Barkhuizen, 2015; Benson, 2018). Narrative inquiry’s capacity of capturing changes and development over time makes it complementary to other research methods (e.g., experiment, observation, and survey; Barkhuizen et al., 2014; Benson, 2021). The strength of narrative inquiry is especially obvious in such fields as language learning and teaching, which often takes a long time (e.g., 47 weeks in this study) and bounds up with broader experiences of both teachers and students (Benson, 2021). Despite only one teacher’s case being involved, we as researchers show that even if it is a single person, he or she can also discover or create new understandings as the time moves along (Corbin and Strauss, 2008; Creswell and Creswell, 2018).

Notably, this study involved the narratives or stories of the first author, CB; therefore, it reconciled both autobiographical and biographical research. For CB, it was autobiographical research; the dual role as both researcher and participant opened up opportunities for intrapersonal and interpersonal communication, but her first-person analysis may also result in a biased stance. For the other two researchers, it was biographical research; they analyzed the data from a third-person perspective, which was complementary to CB’s autobiographical research. In addition, the three researchers’ negotiation of coding results and the comparison and contrast with the findings in the interviews (i.e., spoken narratives), to some extent, worked as triangulation, which enhanced the trustworthiness of this study in turn. In this process, the three researchers, also acting as research tools, made the inner voice of the first author visible to the largest extent through their interpretation of the data (Bao et al., 2016). In other words, our focus was on the depth of the data and nuanced meanings arising from them. Our intention was not to generalize the findings to other populations, but rather to tease out the meanings from them. This is because qualitative research is more interested in finding phenomena to be described in great detail with “thick” or “rich” data for establishing and enhancing trustworthiness (Corbin and Strauss, 2008; see also Dixon et al., 2020).

### Data Collection

**FIGURE 1:**
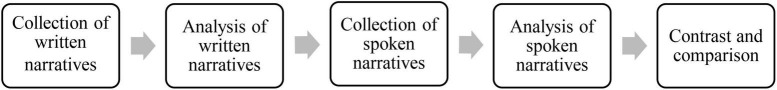
The research process.

The collection and analysis of data were interwoven (refer to Figure 1). The data consist of two parts: CB’s written and spoken narratives. The written narratives involved 26 teaching journals (hereafter, “TJ” or “journal”) which were written by CB during the 47 weeks when she taught this class (refer to Suppelmentary Appendix A). Based on the analysis of written narratives, spoken narratives (Excerpts 1–18) were collected through interviews in which CB retrospectively interpreted the details in the journals and narrated some new perspectives (refer to Suppelmentary Appendix B). All of the data were translated from Chinese into English by the researchers.

### Data Analysis

This study employed thematic analysis, focusing on the content of the participant’s experiences and her reflections on the experiences by searching for themes in the narrative data (Barkhuizen, 2015). Following the process in Figure 1, the written narratives were analyzed first, with both inductive and deductive thematic analysis (Riessman, 2008; Barkhuizen et al., 2014; Miles et al., 2014) through the following steps:

#### Step 1. Analysis of Narratives

The journals, the raw written narratives, were numbered from 1 to 26 in the order in which they were written chronologically. For example, TJ1 was written first, whereas TJ26 was the last. The data of each journal was coded with “week”: if the journal was written during the first week of this program, its time was “Week 1.” According to the topics in each journal, these journals were further segmented into 29 entitled stories (see details in Suppelmentary Appendix A).

Then inductive thematic analysis was employed. From the 29 entitled stories, five common topics emerged: Family A, Family B, the whole class, the institution, and CB’s emotional states, based on which the coding schemes were designed (refer to Table 2). All the 29 stories were coded deductively and manually by the first author and checked by the other two authors, respectively; then the three of us further discussed the results to reach a consensus in the form of discussions of the manuscript in multiple drafts. For example, in Story 1, the sentence “the textbook provided by the program was also Textbook A” indicated that the teacher was forced to use Textbook A by the program, so this sentence was categorized into “the institution”; the sentence “only two families in the class …” introduced the whole class, so it was put in “the whole class”; the information about Family A and Family B was also introduced, which means “Family A” and “Family B” were involved; it can also be detected that “I have never expected” indicated “CB’s emotional states.” As such, the content of Story 1 involved the five topics. Considering the convenience of description, each topic was marked with a capital letter in its column (A, B, C, D, and E), so that such coordinates as “A1” (i.e., Story 1 of Family A) could be used (refer to Suppelmentary Appendix C).

**TABLE 2:** Coding schemes.

Topics		Key words/expressions
Students	Family A	The family or any member of the family e.g., “Family A,” “Mother A,” “Daughter A,” “Son A”
	Family B	The family or any member of the family e.g., “Family B,” “Mother B,” “Father B,” “Daughter B”
Working spaces	The whole class	Something involving or related to both of the two families e.g., “class,” “classroom activities,” “homework,” “the students”…
	The institution	The things or persons related to the institution e.g., “the program,” “the manager,” “Textbook A/B,” “the colleagues”…
CB’s emotional states		(1) The words or expressions about CB’s feelings e.g., “I have never expected,” “surprisingly,” “aggrieved,” “I smiled” … (2) The exclamatory sentences e.g., “What a diligent girl!” (Story 10) (3) CB’s judgment/evaluation of something e.g., “I like them so much” (Story 17)…

However, not all of the content could be categorized so clearly because the roles played by some expressions might be elusive, which required the researchers to perceive, discuss, and clarify what they were or what they meant. For example, when analyzing the sentence in Story 2, “it’s a little bit weird to teach students (without Chinese heritage) how to write ‘
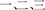
’…,” the researchers held different opinions. The first opinion was that it should belong to “CB’s emotional states” because “the students” were part of CB’s emotions; the second opinion focused on the literal meanings of the words as long as “the students” appeared, it should also be categorized into “the whole class.” Finally, the second opinion was adopted. In addition, the researchers should read between the lines, not overlooking expressed emotions or some other clues, because all the contents were part of a context and were associated with action or inaction (Corbin and Strauss, 2008). For example, no “institution” was mentioned in Story 26, but there was some content still categorized into “institution” (refer to Suppelmentary Appendix C: D26). This is because CB submitted her resignation letter to the institution, from which it could be inferred that the story related to “Resignation Letter” should be categorized under “institution.”

#### Step 2. Narrative Analysis

The fragmented data in each column in Suppelmentary Appendix C were “restoried” into a set of coherent whole ones, each one with an event (e.g., Daughter A’s performance in the Chinese Speech Contest) and a core meaning item (e.g., turning point toward the expression of naturally felt emotions) as its thematic heading or storyline (Polkinghorne, 1995; Consoli, 2021). Then these new stories were interpreted through repeated comparison and contrast between written and spoken narratives.

## Findings and Discussion

CB’s emotional experiences during the 47 weeks were complicated; different emotional states (e.g., frustration, confusion, and happiness) might appear alone or simultaneously in the relationships between CB and the two families.

### CB’s Emotions Related to Family A (RQ 1)

CB’s emotions related to Family A went through two main storylines: “Mother A’s complaint” and “Daughter A’s performance in the Chinese Speech Contest”; the emotional labor strategies she used gradually changed from surface acting to the expression of naturally felt emotions.

#### Storyline 1: Mother A’s Complaint (Surface Acting)

*Mother A complained, because she could not follow me and could learn nothing in the class* (Suppelmentary Appendix C: A3).

This is Mother A’s complaint, which was made in Week 4 (Suppelmentary Appendix A: Story 3), at the very beginning of this program even before CB knew what happened. Therefore, it is not exaggerating to say that Mother A’s complaint was the starting point of CB’s journey in the program. This complaint, to some extent, acted as a catalyst, accelerating the changes not only in CB’s interpersonal communication with students and the institution but also in her intrapersonal communication.

Initially, CB was confused about her relationship with Family A, as she wrote,

*I felt very aggrieved as well as angry. Why didn’t she communicate with me directly? She was indeed active in the class and said thanks to me today*…*What happened?* (Suppelmentary Appendix C: E3).

Then, the manager’s attitude toward Mother A’s complaint made CB’s already-negative emotional state worse:

*What the manager really needed me to do was to make all the students not withdraw from the program; students’ feelings were more important than mine* (Suppelmentary Appendix C: D3).

Students’ complaints told her that she was not as excellent a teacher as she had imagined; the manager’s attitude reminded her that the institution cared more about the students than about her, the teacher.

To respond to such a situation, CB had to behave positively, but she was reluctant and unhappy because her emotions were infused with frustration; therefore surface acting was adopted. Instead of accepting the institution’s arrangement passively and keeping silent (e.g., she was reluctant to use Textbook A but said nothing; Suppelmentary Appendix C: D2, E2), she took action actively to improve her personal teaching materials (e.g., handouts) and communicate positively with the manager. Her effort, in fact, won the manager’s support eventually. As she described:

*I have reorganized all the contents taught in the past four weeks and communicated with the manager (of the program). The manager said these handouts were so amazing. She (the manager) agreed to help me* (Suppelmentary Appendix C: D4).

In addition to the manager’s support, CB’s change brought her another surprise: Mother A made an apology to her and explained why she complained:

*She was so tired recently that she could not help losing her temper. She also promised to tell the manager that it was not my fault*… (Suppelmentary Appendix C: A5).

Despite a satisfactory resolution to the complaint, CB’s happiness still stayed in the layer of surface acting, or rather she felt more confused than relieved, because “hearing this, I really don’t know whether I should be sad or happy…” (Suppelmentary Appendix C: E5).

Then she started her intrapersonal communication—such confusion originated from the discrepancies between her actual and expected selves, which could be scrutinized in two clues. The first clue was her attitude toward Textbook A, which she thought was not a proper choice, as she said, “my feelings were a little bit complicated, but cannot tell what was wrong now” (Suppelmentary Appendix C: E2); Mother A’s complaint further confirmed her such feelings. Therefore, when preparing the handouts, she really hoped “the students will be satisfied with the handouts” (Suppelmentary Appendix C: C4), and what, in fact, comforted her was “she (Mother A) said these handouts were really useful…she could follow me and review after class” (Suppelmentary Appendix C: A5). The other clue could be scrutinized in “I think I can do much better now…If Mother A were in my present class, she would not complain, either …” (Suppelmentary Appendix B: Excerpt 3), which indicated CB’s dissatisfaction with her incapability then. Such discrepancies motivated her to prove herself not a failure to others as well as herself. In her own words, “I do not hope to disappoint anyone, including myself” (Suppelmentary Appendix C: E4).

#### Storyline 2: Daughter A’s Performance in a Chinese Speech Contest (Turning Point Toward the Expression of Naturally Felt Emotions)

The Chinese speech contest was a turning point. Since then, not only did CB gain the trust of Family A, but her career also stepped into a new phase.

In the process of preparation for the contest, Mother A witnessed CB’s devotion to Daughter A’s preparation after class (Suppelmentary Appendix C: A10), so she was sincerely appreciative of the encouragement CB gave to her daughter. In return, Daughter A got third place in the contest, a really good result for a beginning learner. What surprised Mother A the most was that the photo of her daughter was printed in the local newspaper. This inspiring result also meant Family A attained their learning aim—“the children were expected to master more languages” (Suppelmentary Appendix C: A1) and CB satisfied their needs. So “Family A brought a bunch of flowers to me (CB) and expressed their appreciation to me (CB)” (Suppelmentary Appendix C: A12), and CB also received a sense of achievement therein.

As a reward, “after the contest, I (CB) was invited to be the judge the next year” (Suppelmentary Appendix B: Excerpt 10). CB’s teaching also entered a virtuous circle, and the class was more like a big family (e.g., Daughter A also won the best wishes from Family B, as described in Suppelmentary Appendix C: B12). Then it is reasonable to believe that the appreciation CB received from Family A was sincere and the happiness she felt and expressed was her naturally felt emotion.

CB’s such naturally felt emotion was furthered when she was invited to Family A after the contest, which she interpreted as being treated not only as a trustworthy teacher but also as a friend. The family members shared their most precious things with CB: “Mother A showed me children’s photos and told me she was really proud of them…” (Suppelmentary Appendix C: A14), and “Daughter A and Son A showed me the rabbits they raised” (Suppelmentary Appendix C: A15). Mother A also expressed her homesickness to CB:

*She also told me that the furniture and piano were transported from her own country, which made her have more sense of belonging. Although her native language was English, the pronunciation of Kiwi*^2^ *English was much different from hers* (Suppelmentary Appendix C: A14).

After that visit, Mother A would like to communicate with CB more than before as a friend:

*Recent four weeks, Family A have been to the class much earlier than before. Mother A would like to share her happiness and sorrow with me. Perhaps she treated me as her friend now* (Suppelmentary Appendix C: A16).

More surprisingly, Mother A also remembered what CB liked and prepared a special gift for CB. As CB noted,

*Mother A drove to my home and sent me a present which her husband brought from her hometown, because she knew I had been to her country. I unwrapped the present parcel and found three pencils, on which some famous landmarks of her country were printed. I liked them so much* (Suppelmentary Appendix C: A17 & E17).

Mother A’s sharing aroused CB’s empathy. In reality, CB had already treated Mother A as a good friend, unconsciously. As newcomers to New Zealand, they both had much in common to share; as women, they could understand each other sometimes. Although having not seen each other for a long time, CB still regarded Mother A as a good friend, as she said in the interview.

*Since I have already known why she complained and have known more about her life stories, I can understand her now*…*In addition, I am as old as she was then*…*therefore, I can stand in her shoes*… (Suppelmentary Appendix B: Excerpt 7).

Then CB’s emotions were full of understanding and a sense of belonging when she recollected the stories of Family A. Unconsciously, the expression of her emotions with Family A completely changed from surface acting at the beginning to the expression of naturally felt emotions.

### CB’s Emotions Related to Family B (RQ 2)

Compared with Family A, who invited CB into their life, Family B would more like to keep a proper distance from CB, without complaint, but with more respect and politeness. In CB’s recollection,

*Father B and Mother B were very polite and they put more focus on the learning contents, so when they needed something, they would tell me directly. But they did not like to talk about private things too much*… (Suppelmentary Appendix B: Excerpt 12).

Seemingly, Family B did not catch CB’s much attention. However, Family B seized an irreplaceable position in CB’s heart because of the friendliness, kindness, and silent support they provided to CB. Even so, CB’s emotional labor strategies when interacting with Family B also went from surface acting to the expression of naturally felt emotions, which could be sensed through “the negotiation of Family B’s identities” and “the stories Family B shared with CB.” Each of the two stories also went on *via* CB’s interpersonal and intrapersonal communication.

#### Storyline 1: Negotiation of Family B’s Identities (Surface Acting)

The surface acting was employed when the topic “my country” was talked about in class. Since the members of Family B came from different cultural backgrounds (i.e., Father B was from one of the Pacific Island countries; Mother B was from an Asian country; Daughter B was born in New Zealand; Suppelmentary Appendix C: B1), the topic evoked their different responses, despite them being New Zealand citizens.

Father B emphasized that he was a New Zealander, not allowing Mother B to mention his motherland (Suppelmentary Appendix C: B23). With surface acting, CB, as a person from a different culture, chose to respect and keep silent about Father B’s response, although she felt confused then.

In contrast, Mother B, as a first-generation New Zealand citizen immigrating from another country as well as the wife of a husband from a different cultural background, was engaged in the constant process of making sense of the identities of herself and her daughter. As Mother B once asked CB, “Do you think Daughter B looks like a Chinese girl? That’s cool!” (Suppelmentary Appendix C: B21). CB, as a person sharing with Mother B the same identity (i.e., Asians in New Zealand), tried to understand Mother B: her identities were dependent neither on the official passport she held nor on her husband’s ethnicity, but on what she believed; she hoped her daughter looked more like an Asian girl to be more connected to her nation so that she would feel more sense of belonging in her New Zealand family.

Although CB could not stand in the shoes of Family B completely, from Family B’s responses, CB learned: “identity” might be easy for people (e.g., Mother B) who were willing to connect to their motherlands, but it might cause embarrassment for those (e.g., Father B) who would not like to mention their motherlands for various reasons, or for those (e.g., Daughter B) who could not tell which country they belonged to. However, CB admitted that her emotional strategy was still surface acting because her understanding was just a reminder then, reminding her to consider students’ cultural and ethnic backgrounds and to avoid some sensitive topics in class so as not to embarrass students.

#### Storyline 2: Stories Family B Shared With CB (Turning Point Toward the Expression of Naturally Felt Emotions)

CB’s real resonance with Family B was aroused by the stories Family B shared with her in the last lesson:

… *including how they knew each other, how they came to study in New Zealand and became citizens of this country gradually. They expressed that they could understand my experience and respect my choice and wished me everything went well in the future* (Suppelmentary Appendix C: B28).

In this context, CB discovered more common identities they shared: a newcomer to New Zealand, a student from another country, and an employee who got the first job in New Zealand, in addition to the Asian identity shared with Mother B. Surprisingly, CB also found that it was throughout the course that Family B understood her and supported her silently. Instead of expressing to CB directly, it was through their daughter that they transmitted their support to CB. Then CB’s expression of naturally felt emotions was rich in happiness as well as some sense of belonging, especially in the words she used to describe Daughter B:

*Daughter B was the youngest one among the three children and she was very shy. But I liked her very much and I could sense that she loved me too, because she liked to share her happiness and achievement with me, such as her medal and the experience of ordering food; she also made flowers for me. I know those were what she cherished most*… (Suppelmentary Appendix B: Excerpt 12).

This was Daughter B’s image that impressed CB. As a shy girl, Daughter B never expressed her emotions in words, only wearing a smile on her face. When she brought the medal she won in the Taekwondo competition, “her parents told me (CB) that she hoped to show me this medal and to take a photo with me (CB)” (Suppelmentary Appendix C: B13); when she brought a bunch of hand-made paper flowers, her parents shared her eagerness to prepare gifts for CB (Suppelmentary Appendix C: B18). The limited words she said were “I ordered food in Chinese successfully” (Suppelmentary Appendix C: B22), and then her parents continued to share more details (Suppelmentary Appendix C: B22). Sharing and smiling were the two ways Daughter B expressed her feelings, full of love for CB. CB was deeply touched by the little girl; all CB’s love for the little girl, of course, was out of naturally felt emotions, without any surface acting.

## Overall Discussion (RQ3)

Table 3 summarizes the findings of CB’s emotional labor used in the two families. Accordingly, even if her stories with the two families reached happy endings with the expression of her naturally felt emotions, her relationship with both of the families started with the emotions arising out of surface acting.

**TABLE 3:** Summary of findings.

	Family A		Family B	
Storyline 1	Mother A’s complaint		Negotiation of Family B’s identities	
	**CB’s interpersonal communication**	**CB’s intrapersonal communication**	**CB’s interpersonal communication**	**CB’s intrapersonal communication**
	**CB was confused with Mother A’s complaint**, but she responded to Mother A’s complaint by reorganizing her teaching materials: she received Mother A’s apology and won the manager’s support.	Although the ending was satisfactory, CB still felt confused, because: **(1) She was not satisfied with Textbook A;** **(2) She realized the discrepancies between actual and expected selves.**	**She did not understand** Father B, but chose to respect him and kept silent. She tried to understand Mother B, but **she could not stand in her shoes completely.**	She realized it was necessary to take students’ cultural and ethnic backgrounds into consideration.
**Emotional labor strategy**	Surface acting			

**Storyline 2**	**Daughter A’s Performance in Chinese Speech Contest**		**Stories Family B Shared with CB**	

	**CB’s interpersonal communication**	**CB’s intrapersonal communication**	**CB’s interpersonal communication**	**CB’s intrapersonal communication**
	Family A **trusted** CB and **shared life stories** with CB; The institution invited CB to be the judge the next year.	She treated Mother A as her **friend**; She **shared identities** with Mother A; She felt **sense of achievement as well as belonging**.	Family B **shared some life stories** with CB; They **understood and supported** CB silently throughout the course.	She found out more **common identities** with Family B; She realized Family B’s silent support, which made her feel **happiness and sense of belonging**.
**Emotional labor strategy**	Turning points toward the expression of naturally felt emotions.			

### Surface Acting: Four Discrepancies and CB’s Identity as a Newcomer in the Contexts

Table 3 unveiled four main discrepancies emerging from the stories in Storyline 1: CB’s (1) confusion about Mother A’s complaint; (2) dissatisfaction with Textbook A; (3) disappointment with herself; and (4) incapability of understanding Family B’s national identities. In response to the four discrepancies, CB adopted surface acting.

In essence, CB’s these emotional responses were the results of her identity as a newcomer in the contexts (i.e., both New Zealand and the institution), which worked on CB’s emotions through the negotiation of her identities (Pavlenko, 2003; Park, 2012; Fairley, 2020), or rather, the way she viewed and understood herself (Lave and Wenger, 1991). In the new context, she was unfamiliar with anything but was eager to reconstruct her life and enhance her confidence. In the stories of Storyline 1, she cherished this teaching opportunity because it was her first job in New Zealand, and she hoped to learn local teaching methods to look more like a local teacher. However, such eagerness brought a series of problems: First, as a newcomer, she could not judge whether her previous experiences worked effectively in New Zealand. Therefore, whenever she encountered something differentiated from her knowledge, she categorized those new things into new knowledge and persuaded herself to follow the local way. That is why she had no courage to communicate with the manager, even if she had already sensed that Textbook A was not appropriate. Second, as a newcomer, she was not sure of whether her pedagogical practices met students’ needs even when faced with Mother A’s complaint, which was also why she did not dare to argue with Mother A and the manager even if she sensed it was not her fault that led to Mother A’s complaint. Third, as a newcomer, it was the first time for her to teach students with multiple cultural and ethnic backgrounds; therefore, she did not understand why it was not easy for some students to tell their national identities then. These three problems resulted in the loss of her expected identity as a qualified or excellent teacher, which directly led to her disappointment with herself. Therefore, she was still unable to feel happy heartedly even after Mother A had made an apology to her or she had tried to understand Family B’s identities.

### Turning Points Toward the Expression of Naturally Felt Emotions: Mutual Understandings With the Two Families and CB’s Identity as a Qualified Teacher in the New Contexts

In Table 3, the stories in Storyline 2 presented several key words: “trusted,” “shared life stories,” “understood,” and “supported” in CB’s interpersonal communication; “friends,” “shared identities,” “common identities,” “sense of achievement,” “sense of belonging,” and “happiness” in CB’s intrapersonal communication. Accordingly, CB’s mutual understandings with the two families were aroused by the life stories shared with them; these shared stories invited CB into their lives (e.g., they became friends) and helped CB find out more identities she shared with the two families (e.g., a newcomer to New Zealand and an employee who got the first job in New Zealand) so that she and the two families could stand in the shoes of each other and the psychological gap between them was bridged in return.

In addition, CB, as a teacher, made efforts to match her actual and expected self by helping students understand and apply the language she taught to achieve their learning aims (e.g., Daughter A’s Chinese speech contest and Daughter B’s ordering food in Chinese); such efforts helped her find lost identity (i.e., a qualified and excellent teacher) and brought her professional well-being and emotional rewards (e.g., students’ love, kindness, and trust and support; Benesch, 2017; Gkonou and Mercer, 2018; Miller and Gkonou, 2018; Oxford, 2020). These emotional rewards not only endowed CB with a sense of belonging, happiness, a sense of achievement, and confidence or robust self-efficacy in the new contexts but also nourished her future teaching (Dewaele, 2018). Therefore, all her displayed emotions based on mutual understandings and her identity as a qualified teacher were naturally felt emotions.

On the whole, how CB’s emotions developed was determined by her relationship with the students as well as with herself (Dewaele et al., 2018; Benesch, 2020; Li and Liu, 2021). The sound relationship was based on reducing the discrepancies (e.g., Mother A’s complaint and apology and CB’s actual self and expected self) and arriving at mutual understandings (i.e., trust, responsiveness, and empathy; e.g., her shared identities with Mother A and Family B; Gkonou and Mercer, 2017; Benesch, 2020).

**FIGURE 2:**
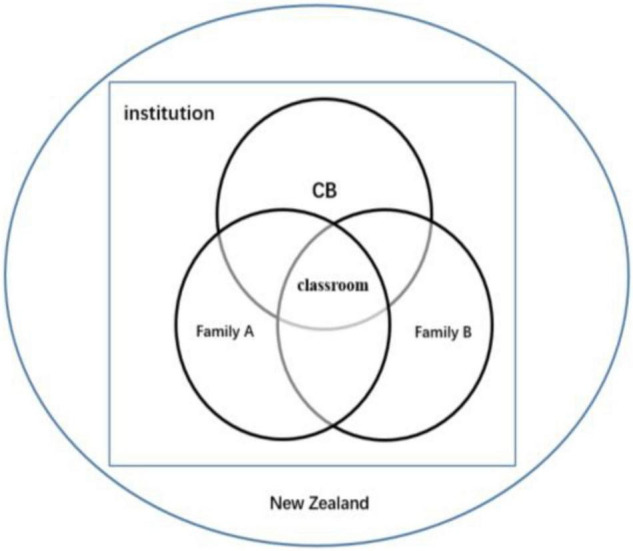
The relationship between CB and other factors.

However, it should be noticed that both discrepancies and mutual understandings were just phenomena that were influenced by two bigger layers of the contexts: the working context (i.e., the institution) and the social context (i.e., New Zealand; Miller and Gkonou, 2018; refer to Figure 2). The contexts were like a theater, inside which CB and the two families were the main characters displaying the stories. All the characters not only interacted with each other and with the characters they played (see the stories in *Findings*) but also interacted with the theater environment (Miller and Gkonou, 2018). Within a theater with the main aim of earning money or with an atmosphere of isolating newcomers, therein the only role teachers could act as was a teacher who employed surface acting to respond to emergent issues. In contrast, within a theater that was full of love, inclusiveness, and understanding, teachers could have a sense of belonging to explore their potential to create and perform their characters better; once teachers appreciated and trusted the characters they played, their emotions would be expressed naturally and heartedly.

## Conclusion

The findings revealed that the teacher’s emotions and choices of emotional labor strategies were highly connected to her identity and to her relationship with students. Such connections further prove that any person’s life is unique, complex, and unpredictable when he/she is embedded in and exposed to complex social milieus (e.g., values and ideologies; Consoli, 2021); these social milieus shape one’s self (Ahn, 2021) and influence one’s emotions; in return, the emotions expressed and experienced by the person enable him/her to navigate various situations and cultures and guide him/her to make decisions and establish and maintain relationships with others (Ahn, 2021), which are significant aspects of teachers’ work no matter what types of teaching contexts they are embedded in.

In terms of the methodology, this study reconciled the perspectives held by the two camps related to emotional labor, namely, structural and post-structural camps. Employing the perspectives held by the former camp, it took the two main dimensions or strategies (i.e., surface acting and the expression of naturally felt emotions) raised by Diefendorff et al. (2005) as its guide to explore how the CAL teacher exercised her emotional labor in a new teaching context. Adopting narrative inquiry, a type of qualitative method advocated by the latter camp, it analyzed the details and trajectories of how the social contexts influenced the CAL teacher’s choices of emotional labor strategies.

In addition, the focus of this study is on a family-based teaching context, which complements the existing research based on the mainstream CAL programs or online platforms. The data from this study and the analysis process also provide CAL teachers or other language teachers with the experiences of (1) how to communicate, and reconstruct the relationship, with themselves in new contexts; (2) how to use their own experiences to nourish, and connect with, their past and future selves; (3) how to be a teacher in a context that is different from stereotypical teaching environments; (4) how to balance students’ needs and institutional requirements; and (5) how to manage a class with students from different ethnic groups and/or of different ages. The findings also share with the institutions an idea about what kind of working atmosphere they can create for teachers, especially for novice teachers.

Admittedly, the findings from one case cannot be generalized; however, the process of how her emotions developed through the interaction with students and herself provides a model for other teachers who are working or will work in similar contexts.

## Data Availability Statement

The original contributions presented in the study are included in the article/Supplementary Material, further inquiries can be directed to the corresponding author.

## Ethics Statement

The studies involving human participants were reviewed and approved by The University of Auckland Human Ethics Committee. The patients/participants provided their written informed consent to participate in this study.

## Author Contributions

CB conceived and designed the study, collected and analyzed the data, and wrote the first draft of the manuscript. LZ and HD revised the manuscript. All authors agreed to the final version before LZ got it ready for submission as the corresponding author.

## Conflict of Interest

The authors declare that the research was conducted in the absence of any commercial or financial relationships that could be construed as a potential conflict of interest.

## Publisher’s Note

All claims expressed in this article are solely those of the authors and do not necessarily represent those of their affiliated organizations, or those of the publisher, the editors and the reviewers. Any product that may be evaluated in this article, or claim that may be made by its manufacturer, is not guaranteed or endorsed by the publisher.

## Footnotes

1 Based on https://nzhistory.govt.nz/culture/history-of-new-zealand-1769-1914.

2 Kiwi: the way New Zealanders call themselves.
