# Supplementary material for: Emotional Labor in Teaching Chinese as an Additional Language in a Family-Based Context in New Zealand: A Chinese Teacher’s Case
Source: Front Psychol. 2022 Jun 15;13:902700. doi: 10.3389/fpsyg.2022.902700 (PMC9240204; doi:10.3389/fpsyg.2022.902700)
Supplement: Supplementary file 1 [file Data_Sheet_1.pdf]

SUPPLEMENTARY APPENDICES---A, B, C

Appendix A. Summary of CB's Teaching Journals (TJ)

TJ	Week	Summary
1	1	**Story 1 A brief introduction to the Class**<br>Today is the first day that I work for the program. The textbook provided by the program was also Textbook A, the same as the one used for students with Chinese heritage. I have never expected that my class is so special: only two families in the class: Family A (Mother A, Daughter A and Son A) is from an English-speaking country; Family B (Father B, Mother B and Daughter B) is a multiple cultural family (Father B is from one of Pacific Island countries; Mother B is from an Asian country; Daughter B was born in New Zealand).<br>They shared with me the reasons for learning Chinese: in Family A, the children were expected to master more languages; in Family B, the father needed to communicate with Chinese people on business.
		**Story 2 Textbook A? Perhaps, no!**<br>The first class has been over, which seemingly went on smoothly; however, I still have sensed that my feelings were a little bit complicated, but cannot tell what was wrong now… Perhaps, it's a little bit weird to teach students (without Chinese heritage) how to write "一、二、三" （Chinese characters for numbers "1,2,3" respectively） , but the textbook (Textbook A) shows it. In addition, which language should I use in class? English? Chinese? I have asked colleagues, but no one has ever taught this type of class, so I have got no answer…
2	4	**Story 3 Why did she complain? I have no idea.**<br>The manager told me that I had been complained by Mother A, because she could not follow me and could learn nothing in the class. I felt very aggrieved as well as angry, "Why didn't she communicate with me directly? She was indeed active in the class and said thanks to me today… what happened?"<br>It was also the moment that I realized what the manager really needed me to do was to make all the students not withdraw from the program…Students' feelings were much more important than mine…
3	5	**Story 4 I don't want to make anyone disappointed, including myself.**<br>Having worked for so many years, I do not hope to disappoint anyone, including myself. I really cannot accept such complaints. Therefore, I have reorganized all the contents taught in the past four weeks and communicated with the manager (of the program). On the one hand, I needed to show my efforts and let her know what I have taught; on the other hand, I hoped that she could help me print out the handouts for each class in advance. The manager said these handouts were so amazing. Happily, she agreed to help me. Really hope the students will be satisfied with the handouts.
4	6	**Story 5 Why did she complain? I know now.**<br>Surprisingly, Mother A made an apology to me today. She told me why she made complaint: she was so tired recently that she could not help losing her temper. She also promised to tell the manager that it was not my fault…Hearing this, I really don't know whether I should be sad or happy…Anyway, she said these handouts were really useful…she could follow me and review after class…

5	7	**Story 6 Textbook B! Good!**<br>This week, Textbook B was used. I think it is much better than Textbook A, because its content is more suitable for non-native Chinese speakers. With this one, I can teach much easily than before. Apparently, students liked this textbook much more. Perhaps, I can be more relaxed.
6	8	**Story 7 Yes! An idea occurred to me! I know how to organize the class effectively.**<br>Today, I asked the three children to come to the whiteboard to write some simple Chinese characters and do some exercises. All of the children and their parents were happy. I seem to have found out an effective way to organize classroom activities: to design some activities for the three children and encourage the parents to act as coaches to help the children…As such, all of them will be active and happy; the atmosphere of the class also started to become much more relaxing…Happy!
7	9	**Story 8 A new type of homework: Role Play**<br>Today, the homework is "Role Play". Really hope the family members can cooperate well. I have also found that among all the six students, Father B's pronunciation was the best, especially the four tones he pronounced.
8	12	**Story 9 Notice: Chinese Speech Contest**<br>The program will organize a Chinese Speech Contest in two weeks. I shared the information with the two families, hoping them participate in it. In Family A, Mother A suggested that Daughter A should have a try, and Daughter A agreed immediately. In Family B, Mother B said that she looked like a native Chinese speaker but could not speak Chinese, which made it strange if she attended the contest. Therefore, she encouraged Father B to participate, but he refused, because he thought it was like children's activity.
9	13	**Story 10 Daughter A, come on!**<br>Daughter A has brought a monologue she has prepared for the Chinese Speech Contest. After the class, she read it to me. Because she only had learnt Chinese for only three months, it was a brief self-introduction, including all she had learnt. It was amazing that she worked so hard in the past week. Indeed, she has made a huge progress...I proofread her manuscript and read it and correct her pronunciation word by word. Mother A used her cellphone to record my reading and told Daughter A to practice more and correct her pronunciation at home. What a diligent girl!
10	14	**Story 11 Daughter A, congratulations!**<br>In Chinese Speech Contest, Daughter A got the third place. Well done!
11	15	**Story 12 "Thank you, Ms. CB!" Family A said.**<br>Family A brought a bunch of flowers to me and expressed their appreciation to me. What surprised me more was that the photo of Family A and I, which was taken during the contest, was printed in local newspaper. Family B were also happy and said congratulations to Daughter A.
12	16	**Story 13 "This is my medal." Daughter B showed me.**<br>I am so happy that Daughter B brought a bronze medal she won in Taekwondo competition. Her parents told me that she hoped to show me this medal and to take a photo with me. What a lovely girl! Family A also applauded for her. How happy I am! Our class is like a big family more and more.
13	20	**Story 14 Mother A's sense of belonging**<br>I visited Family A's home. Mother A showed me the children's photos and told me she was

		really proud of them. She also told me that the furniture and piano were transported from her own country, which made her have more sense of belonging. Although her native language was English, the pronunciation of Kiwi English was much different from hers.
		**Story 15 Look! This is my rabbit!**<br>Daughter A and Son A showed me the rabbits they raised. One person, one rabbit. Each person should be responsible for his/her own rabbit. It is really a smart way to cultivate children's sense of responsibility.<br>Through this visit, I think I have learnt a lot. I am also inspired to know more about how to educate children in different countries.
14	24	**Story 16 More communication in the class**<br>Recent four weeks, Family A have been to the class much earlier than before. Mother A would like to share her happiness and sorrow with me. Perhaps she treated me as her friend now. In Family B, the parents also encouraged Daughter B to use Chinese to share stories with me, telling me how they had practiced at home. The parents from the two families also liked to communicate and to talk about children's education. I enjoyed the class time more and more.
15	26	**Story 17 The present from Mother A**<br>This week is vacation. Mother A drove to my home and sent me a present which her husband brought from her hometown, because she knew I had been to her country. I unwrapped the present parcel and found three pencils, on which some famous landmarks of her country were printed. I liked them so much.
16	27	**Story 18 The most valuable paper flowers! Thank you so much, Daughter B!**<br>This was the first class after vacation. Daughter B brought me a bunch of flowers. How beautiful they are! I was surprised to know that these were hand-made flowers, which were made by Daughter B during the vacation. Her parents told me she learnt how to make them and prepared for me. I was really moved by the little girl!
17	32	**Story 19 Back to Textbook A**<br>Having learnt Chinese about 8 months, students have mastered many useful Chinese expressions in daily life. This week, students have been encouraged to use Textbook A together with Textbook B. It's time to recognize and try to write more Chinese characters. Now they would like to write much more.
18	34	**Story 20 Ms. CB, could you please speak more Chinese in class?**<br>Today, Father B asked me, "Ms. CB, could you please speak more Chinese in class? Then I can pick up more expressions." I asked other students, "How about you? May I speak more Chinese?" All of them said yes. That's a good idea! I will do it immediately.
19	35	**Story 21 "Do you think my daughter looks like a Chinese girl?" Mother B asked.**<br>Mother B always kept silent in class.<br>Today she asked me, "Do you think my daughter looks like a Chinese girl?"<br>I replied, "Yes, indeed!"<br>Mother B said happily, "That's cool!"<br>Although I don't know why she asked this, this short conversation made me feel that we became much closer than before; perhaps, both of us were from Asian countries and she hoped her daughter looked more like an Asian girl.
20	37	**Story 22 "I successfully ordered food in Chinese." Daughter B**<br>Today, when Family B came into the classroom, Daughter B was so excited. She told me, "I

		ordered food in Chinese successfully." Then, her parents shared with me more details about what happened in a Chinese restaurant, "Daughter B wanted to order food by herself." I smiled with them.
21	38	**Story 23 "I am Kiwi," Father B said.**<br>Today, the topic in our class was "My country". We tried to use Chinese to introduce our countries as much as possible. I described China and the three persons in Family A introduced their country actively and excitedly. When it was Family B's turn, Mother B introduced her country, and then said Father B was from ***(the country name). Father B interrupted her, "No, I am Kiwi. I am a New Zealander." Mother B did not mention the country any more.
22	39	**Story 24 Who am I? Who should I be?**<br>Father B's thought about which country he belonged to also reminded me to think about the question carefully: When I am standing among the students from various cultural background, who should I be? A newcomer of New Zealand? A university student? Just a teacher teaching Chinese? A friend? A Chinese person? …Perhaps, that's not an easy question.
23	40	**Story 25 What does "success" mean?**<br>Today, the manager of the program told me that my class was a successful one, so the program would enroll more families in the future. I cannot tell what "success" means for a class, but the class is a harmonious one indeed. I am surprised to find that I am so peaceful when hearing these words.
24	43	**Story 26 Resignation Letter**<br>Today, I have submitted my Resignation Letter, expressing that I am leaving New Zealand in one month for academic purpose. Having taught these students for about ten months, I feel a little bit sad.
25	44	**Story 27 Family A is leaving**<br>Today, Mother A told me that their family would leave New Zealand temporarily because of her job, so she came here to attend the last lesson. She also liked to say thanks to me, because her children started to love to learning Chinese, which brought me a sense of achievement. We talked a lot after class… (For the reason of Family A's privacy, the details have not been written here).
26	47	**Story 28 The last lesson**<br>This is the last lesson I taught for this program, only Family B and I. Father B and Mother B shared with me their stories, including how they knew each other, how they came to study in New Zealand and became citizens of this country gradually. They expressed that they could understand my experience and respect my choice and wished me everything went well in the future.
		**Story 29 My reflection**<br>Family B did not communicate with me much after class, but I have also noticed that they have been learning diligently. Among all the six persons in this class, Father B's Chinese language proficiency is the highest and his pronunciation is the best one. I have also noticed that Family B has never mentioned Daughter B's academic grades; what they have shared with me most is about the some interesting anecdotes in daily life.

Appendix B. Summary of CB's Interviews

Relevant Stories	Questions	Excerpts of CB's Replies
1	If you were the teacher of this type of class now, what would you do in the first class?	**Excerpt 1**<br>Now, when I meet my students for the first time, I would prepare a questionnaire in advance and get familiar with students' background and learning aims as soon as possible.
2 & 6	You have mentioned textbooks.<br>If you were the teacher of this class now and you were forced to use Textbook A, how would you use it?	**Excerpt 2**<br>Now, I have much more teaching experience now, I would tell the manager of the organization directly that Textbook A is not a proper choice and advise to use Textbook B. If I must use Textbook A, I would add more contents by myself. For example, when I teach numbers, I would teach students how to use these numbers, such as expressing time and price, rather than focus on how to write these Chinese characters.
3	Now, what would you do to deal with students' complaints, such as Mother A's complaint?	**Excerpt 3**<br>I think I can do much better now…Perhaps my students would not complain…LOL. If Mother A were in my present class, she would not complain, either…LOL Just kidding. If some student complains, I will investigate the reasons for the complaint: if it is because I cannot satisfy his/her needs in the class, I will try to adjust my teaching plan and try to provide what he/she needs; if it is not because of me, perhaps what I can do is only to remind myself "that is life"…LOL
4	Why were you afraid of making other people disappointed?	**Excerpt 4**<br>Actually, I was also afraid to make myself disappointed then. Before I went to New Zealand, I had worked for many years, but I gave up the job and chose to study in NZ. Therefore, I must prove that my choice (to study in NZ) was correct then. In addition, I did not have scholarship then, which means my economic pressure was very big…This program was the first job I got in NZ, so I should seize the opportunity to prove that I could survive in NZ…I was afraid to lose the job then…<br>Another reason is, perhaps, a little bit exaggerated... But I still believe that as a CAL teacher, I still think I am an important Chinese person students can contact; through me, they can form an idea of what Chinese people are like…To some extent, I represent the image of Chinese people in students' eyes. So I will not disappoint my students; I don't want to leave any bad impression on students, let alone making students disappointed with Chinese people…Perhaps, that's my responsibility.
	Do you still care about whether other people	**Excerpt 5**<br>Yes, I always care about it, but not as much as I did before, because

	would be disappointed or not?	I know what I am doing much more clearly now...I will focus more on how to do the job better than on what others' feeling…Of course I will communicate with them, and will not make students disappointed.
	Why?	**Excerpt 6**<br>Because that time, I was much younger. I always wanted to prove that I was excellent and I wanted to do everything best. But now I have more confidence, on the one hand; on the other hand, I know I am just an average person who has advantages, disadvantages and limitations so that I cannot satisfy all the students' needs perfectly even if I am a teacher and I would like to try my best …
**5**	What are your present attitudes towards Mother A's complaint?	**Excerpt 7**<br>Since I have already known why she complained and have known more about her life stories, I can understand her now…In addition, I am as old as she was then…therefore, I can stand in her shoes …
7-8	How did you organize your class then?	**Excerpt 8**<br>It depended on the specific teaching contents of each class. For example, when activities were needed, I would like to design some activities for children; then the parents acted as coaches to encourage and help their children to complete… or just acted as audience to watch and applaud for their children. When we needed to talk about some ideas, such as the topic related to cultures, parents would communicate more…
	Do you still think these activities were effective?	**Excerpt 9**<br>Yes, of course.
9-12	Could you describe what role the Chinese Speech Contest played in your teaching?	**Excerpt 10**<br>To some extent, it was a turning point. Of course, as a teacher, I got a sense of achievement. At the same time, both the manager and Mother A expressed their appreciation for my devotion…Actually, after that contest, I was invited to be the judge the next year…. Family B also expressed their best wishes to Daughter A…the class was more like a big family.
14-17	How did you sense Mother A's attitude to you had changed indeed?	**Excerpt 11**<br>It was also after the competition that Mother A liked to communicate with me more than before. Perhaps we had worked together to help her daughter win the competition, or she started to treat me as her friend. You can see, she also invited me to her home and prepared some special presents for me...(see Stories 14-17)
13 & 18 & 20 & 22	In your stories, you did not mention Family B as much as Family A. Does it mean that you prefer Family A more than Family B?	**Excerpt 12**<br>I don't think so. Although I wrote Family A more than Family B, I paid as much attention to Family B in class. Perhaps after class, I communicated with Family A more, because Mother A was more talkative. Father B and Mother B were very polite and they put

		more focus on the learning contents, so when they needed something, they would tell me directly. But they did not like to talk about private things too much (e.g., Story 20). But they did not like to talk about private things too much. Daughter B was the youngest one among the three children and she was very shy. But I liked her very much and I could sense that she loved me too, because she liked to share her happiness and achievement with me, such as her medal and the experience of ordering food; she also made paper flowers for me. I know those are what she cherished most…
12& 21& 23&28	Did you try to communicate with Family B privately?	**Excerpt 13**<br>Yes, actually Mother B talked with me a little bit more. Perhaps both of us were from Asian countries and had more in common.
	What did you talk?	**Excerpt 14**<br>Sorry, that's Family B's privacy. I won't share here.
25	If you did not leave NZ for academic purpose then, would you continue to teach the class?	**Excerpt 15**<br>Perhaps, yes. But I knew I would leave sooner or later, because I could not stay in NZ forever. Actually, no one could stay in one place forever. Even if I did not leave, the students, such as Family A, would leave someday later for various reasons. They attended voluntarily; they also withdrew voluntarily. So just cherish the times we stayed together…Ah, I am missing them now…
24 & 29	You wrote several stories related to your reflection. Do you still keep the habit of reflection?	**Excerpt 16**<br>Yes. I have not kept the habit on purpose, but I have realized that I always do it. I always think if I had a chance to go back to the past, what I would do. Perhaps reflection is part of my life…
	If you could have a conversation with yourself in the past, what would you say?	**Excerpt 17**<br>Don't be afraid. Everything will be better.
	If you could have a conversation with yourself in the future, what would you say?	**Excerpt 18**<br>I still don't know what you have experienced, but I also hope you are a happy person and you are satisfied with every choice I have made now.

Appendix C. Coded Stories in CB's Teaching Journals

	A	B	C	D	E
Stories	Family A	Family B	The whole class	The institution	CB's emotional states
1	Mother A; Daughter A; Son A; from an English-speaking country; the children were expected to master more languages.	Father B was from one of pacific Island countries; Mother B was from an Asian country; Daughter B was born in NZ; the father needed to communicate with Chinese people on business.	…my class is so special.	The textbook provided by the program was also Textbook A, the same as one the one used for students with Chinese heritage.	I have never expected that …
2	***	***	The first class has been over, which seemingly went on smoothly; students (without Chinese heritage)	The textbook (Textbook A) shows it; I have asked colleagues, but no one has every taught this type of class…	I still have sensed that my feelings were a little bit complicated, but cannot tell what was wrong now; it's a little bit weird to …; I have got no answer.
3	Mother A complained, because she could not follow me and could learn nothing in the class. She (Mother A)was active in the class and said thanks to me today.	***	***	The manager told me that I had been complained by Mother A.<br>What the manager really needed me to do was to make all the students not withdraw from the program; students' feelings were more important than mine.	I felt very aggrieved as well as angry. Why didn't she communicate with me directly? She was indeed active in the class and said thanks to me today…what happened?<br>It was also the moment that I realized…
4	***	***	Really hope the students will be satisfied with the handouts.	I have reorganized all the contents taught in the past four weeks and communicated with the manager (of the program).<br><br>The manager said these handouts were so amazing.<br>She (the manager) agreed to help me.	I do not hope to disappoint anyone, including myself; I really cannot accept such complaints. On the one hand, I needed to show my efforts and let her (the manager) know what I have taught; on the other hand, I hoped that she could help me print out the handouts for each week in advance. Happily, …

5	Mother A made an apology to me today. She told my why she made complaint: she was so tired recently that she could not help losing her temper. She also promised to tell the manager that it was not my fault; she said these handouts were really useful…she could follow me and review after class.	***	***	the manager	Surprisingly…;<br>Hearing this, I really don’t know whether I should be sad or happy…
6	***	***	Apparently, students liked this book much more.	Textbook B was used.	Perhaps, I can be more relaxed.
7	***	***	I asked the three children to come to the whiteboard…;<br>All of the children and their parents were happy…all of them will be active and happy; the atmosphere of the class also started to become much more relaxing.	***	Happy!
8	***	Father B’s pronunciation was the best, especially the four tones he pronounced.	the homework is “Role Play”.	***	Really hope the family members can cooperate well…I have also found (that among the six students, Father B’s pronunciation was the best…)
9	Mother A suggested that Daughter A should have a try, and Daughter A agreed immediately.	Mother B said that she looked like a native Chinese speaker but could not speak Chinese, which made it strange if she attended the contest.	I shared the information with the two families.	The program will organize a Chinese Speech Contest in two weeks.	…hoping them participate in it.

		Therefore, she encouraged Father B to participant, but he refused, because he thought it was like children's activity.			
10	Daughter A has brought a monologue she has prepared for the Chinese Speech Contest. After the class, she read it to me and I corrected her pronunciation word by word…She worked so hard in the past week. Indeed, she has made a huge progress…Mother A used her cellphone to record my reading and told Daughter A to practice more and correct her pronunciation at home.	***	***	***	It was amazing… what a diligent girl!
11	Daughter A got the third place.	***	***	***	Well done!
12	Family A brought a bunch of flowers to me and expressed their appreciation to me.	Family B were also happy and said congratulations to Daughter A.	***	***	What surprised me more was that the photo of Family A and I, which was taken during the contest, was printed in local newspaper.
13	Family A also applauded for her (Daughter B).	…Daughter B brought a bronze medal she won in Taekwondo competition. Her parents told me that she hoped to show me this medal and to take a photo with me.	Our class is like a big family more and more.	***	I am so happy…<br>What a lovely girl!

14	I visited Family A's home. Mother A showed me the children's photos and told me she was really proud of them. She also told me that the furniture and piano were transported from her own country, which made her have more sense of belonging. Although her native language was English, the pronunciation of Kiwi English was much different from hers.	***	***	***	***
15	Daughter A and Son A showed me the rabbits they raised. One person, one rabbit. Each person should be responsible for his/her own rabbit.	***	***	***	It is really a smart way…; I am also inspired to know more about how to educate children in different countries.
16	Recent four weeks, Family A have been to the class much earlier than before. Mother A would like to share her happiness and sorrow with me. Perhaps she treated me as her friend now.	In Family B, the parents also encouraged Daughter B to use Chinese to share stories with me, telling me how they had practiced at home.	The parents from the two families also liked to communicate and to talk about their children's education.	***	I enjoyed the class time more and more.
17	Mother A drove to my home and sent me a present which her husband brought from her hometown, because she knew I	***	***	***	I unwrapped the present parcel and found three pencils, on which some famous landmarks of her country were printed. I like them so much.

	had been to her country.				
18	***	Daughter B brought me a bunch of hand-made paper flowers… which was made by Daughter B during the vacation. Her parents told me she learnt how to make them and prepared for me.	***	***	How beautiful they (the flowers) are!<br>I was really moved by the little girl!
19	***	***	Having learnt Chinese about 8 months, students have mastered many useful Chinese expressions in daily life. This week, students have been encouraged to use Textbook A together with Textbook B…Now they would like to write much more.	…students have been encouraged to use Textbook A together with Textbook B.	***
20	***	Father B asked me, "Ms. CB, could you please speak more Chinese in class? Then I can pick up more expressions."	I asked other students…All of them said yes.	***	That's a good idea!
21	***	Mother B always kept silent in the class. Today she asked me, "Do you think Daughter B looks like a Chinese girl?"…Mother B said happily, "That's cool!"	***	***	Although I don't know why she asked this, this short conversation made me feel that we became much closer than before, perhaps, both of us were from Asian countries and she hoped her daughter looked more like an Asian.
22	***	Today, when Family B came into the class, Daughter B was so excited. She told me, "I ordered food in Chinese successfully." Then, her	***	***	I smiled with them.

		parents shared with me more details... "Daughter B wanted to order food by herself."			
23	Family A introduced their country actively and excitedly.	Mother B introduced her country, and then said Father B was from (the country name). Father B interrupted her, "No, I am Kiwi. I am a New Zealander." Mother B did not mention the country any more.	The topic in our class was "my country".	***	***
24	***	Father B's thought about which country he belonged to…	…students from various countries…	***	(Father B's thought about which country he belonged to) reminded me to think about the question carefully…who should I be?. A newcomer of New Zealand? A university student? Just a teacher teaching Chinese? A friend? A Chinese person? Perhaps, that's not an easy question.
25	***	***	***	Today, the manager of the program told me that my class was a successful one, so the program would enroll more families in the future.	I cannot tell what "success" means for a class, but the class is a harmonious one indeed. I am surprised to find that I am so peaceful when hearing these words.
26	***	***	***	Today, I have submitted my Resignation Letter, expressing that I am leaving New Zealand in one month for academic purpose.	Having taught these students for about ten months, I feel a little bit sad.
27	Today, Mother A told me that their family would leave NZ temporarily because of her job, so she came here to	***	***	***	(She also liked to say thanks to me, because her children started to love learning Chinese), which brought me a sense of achievement.

	attend the last lesson. She also liked to say thanks to me, because her children started to love learning Chinese…				
28	***	This is the last lesson I taught for this program, only Family B and I were here. Father B and Mother B shared with me their stories, including how they knew each other, how they came to study in New Zealand and became citizens of this country gradually. They expressed that they could understand my experience and respect my choice and wished me everything went well in the future.	***	***	
29	***	Family B did not communicate with me much after class, but I have also noticed that they have been learning diligently. Among all the six persons in the class, Father B's Chinese language proficiency is the highest and his pronunciation is the best one. I have also noticed that Family B has never mentioned Daughter B's academic grades; what they have shared with me most is about some interesting anecdotes in daily life.	***	***	I have also noticed that they have been learning diligently…
